# Case Report: EAdi-guided ventilatory management leads to successful extubation in a pediatric myasthenic disorder

**DOI:** 10.3389/fped.2026.1893086

**Published:** 2026-10-14

**Authors:** Marjet J. A. M. Braamskamp, Alette A. Koopman, Martin C. J. Kneyber

**Affiliations:** 1 Division of Pediatric Critical Care Medicine, Department of Pediatrics, Beatrix Children’s Hospital, University Medical Center Groningen, Groningen, Netherlands; 2 Critical Care, Anaesthesiology, Peri-Operative & Emergency Medicine (CAPE), University of Groningen, Groningen, Netherlands

**Keywords:** electrical activity of the diaphragm (EAdi), extubation, mechanical ventilation weaning, neuromuscular respiratory failure, pediatric intensive care unit

## Abstract

Monitoring of diaphragmatic electrical activity (EAdi) may provide real-time insight into neural respiratory drive, but data in pediatric myasthenic disease are scarce. We report a 2-year-old girl requiring mechanical ventilation for respiratory failure due to an initial diagnosis of myasthenic syndrome. Following initiation and escalation of pyridostigmine, median EAdi rose from 1.69 to 7.7 µV while ventilatory support was simultaneously reduced, resulting in successful extubation after 5 days. This case illustrates the potential added value of EAdi monitoring alongside conventional ventilator parameters in tracking neuromuscular recovery during weaning.

## Introduction

The risk of unrecognized respiratory muscle weakness in patients with neuromuscular disorders underscores the need for objective monitoring of respiratory muscle function to personalize ventilatory support and guide decision-making regarding the weaning process. Monitoring the electrical activity of the diaphragm (EAdi) provides real-time insight into neural respiratory drive and is increasingly used in the Pediatric Intensive Care Unit (PICU). However data regarding its application in pediatric myasthenic disease are scarce (1–3).

## Case description

We present a case of a previously healthy 2-year-old girl (13 kg) born from a mother of Dutch-Caribbean origin and a father of Dutch descent who presented with acute respiratory failure requiring mechanical ventilation. Her past medical, psychosocial and family history were unremarkable, with normal growth and achievement of developmental milestones, including walking and speech. Family history reported a paternal uncle with Duchenne dystrophy and no consanguinity.

She presented with a one-week history of progressive fatigue, coryzal symptoms, and persistent cough. On examination, she was lethargic and pale, with marked tachycardia and tachypnea. Oxygen saturation was 87% on a non-rebreather mask (15 L/min; FiO_2_ 1.0) and pCO_2_ in arterial blood gas was 8.6 kPa, prompting emergency endotracheal intubation and subsequent mechanical ventilation in the PICU. Chest x-ray showed possible infiltrative abnormalities. Empiric antibiotics were initiated for suspected viral or bacterial pneumonia; however, infection parameters (CRP, leukocyte counts) were low and blood and sputum cultures remained negative. A spontaneous breathing trial on day 2 using FiO_2_ 0.50 and 5 cm H2O continuous positive airway pressure (without inspiratory pressure augmentation) failed due to unexpected neuromuscular weakness.

## Diagnostic assessment

Additional medical history revealed that the patient had developed progressive bilateral ptosis in the preceding 2 months, for which she compensated by head tilting and eyebrow raising. Other symptoms included increased daytime drooling and fatigability after exertion. In recent weeks, dysphagia presented as prolonged chewing and food avoidance, while speech articulation and fine motor development remained age-appropriate. No previous episodes of respiratory insufficiency or muscle weakness had been reported. The constellation of progressive ptosis, bulbar symptoms, and exercise-induced fatigability was suggestive of a myasthenic syndrome. Small-fiber electromyography supported this diagnosis. Initial acetylcholine receptor antibody testing and anti-muscle specific kinase antibodies during PICU admission were negative.

## Therapeutic interventions

Pyridostigmine was initiated with dose escalation after 36 h. To gain more insight into respiratory muscle activity before and after treatment initiation, structured analysis of ventilatory parameters was performed 60 h before pyridostigmine initiation until 120 h thereafter. In addition to conventional ventilatory parameters including peak inspiratory pressure (Ppeak) and tidal volume, continuous monitoring of EAdi was performed using a specialized esophageal electrode catheter. Ppeak was defined as the maximum airway pressure, measured by the ventilator during inspiration.

The respiratory monitoring started at *t =* −60 h, where *t* = 0 denotes initiation of pyridostigmine and *t* = 36 h denotes dose escalation as shown in Table 1. In the 6 h preceding treatment initiation (*t* = 0 h) the median EAdi was 1.69 [1.25–2.86] µV, Ppeak was 15.8 [15.3–24.2] cm H_2_O and tidal volume was 5.97 [5.37–6.44] mL/kg under ventilator settings of pressure control 14 cm H_2_O, positive end- expiratory pressure (PEEP) 6 cm H_2_O and FiO_2_ 30%. Following pyridostigmine initiation and dose escalation, median EAdi increased to 7.7 [5.12–11.07] µV in the 6 h preceding extubation with reduced ventilator settings to pressure control 10 cm H_2_O, PEEP 5 cm H_2_O and FiO_2_ 25%. The time course of EAdi, tidal volume, and Ppeak in relation to pyridostigmine treatment is shown in Figure 1.

**Table 1:** CARE timeline of the clinical course and relation to EAdi monitoring and pyridostigmine treatment.

Time point	Relation to EAdi analysis/pyridostigmine	Clinical status/symptoms	Diagnostic assessment/findings	Intervention/respiratory course
2 months before PICU admission	Before EAdi monitoring	Gradual onset of bilateral ptosis, compensated by head tilting and eyebrow raising	—	—
Weeks before admission	Before EAdi monitoring	Increasing daytime drooling, fatigability after exertion, and dysphagia with prolonged chewing and food avoidance	—	No previous episodes of respiratory insufficiency or generalized muscle weakness had been reported
1 week before admission	Before EAdi monitoring	Progressive fatigue, coryzal symptoms, and persistent cough	—	—
Day 0 (PICU admission)	Before EAdi analysis/before pyridostigmine	Lethargy, pallor, marked tachycardia, tachypnea, and hypoxemia despite a non-rebreather mask	Arterial blood gas showed hypercapnia (pCO_2_ 8.6 kPa). Infection markers were low, and blood and sputum cultures remained negative	Acute endotracheal intubation and initiation of invasive mechanical ventilation; empiric antibiotics started for suspected viral or bacterial pneumonia
Day 2	Immediately before start of the structured EAdi analysis window	Spontaneous breathing trial failed because of unexpected neuromuscular weakness	Additional history revealed progressive ptosis, bulbar symptoms, and exertional fatigability, raising suspicion of a neuromuscular transmission disorder. Single-fiber electromyography supported this diagnosis. Initial acetylcholine receptor antibody testing was negative	Spontaneous breathing trial with FiO_2_ 0.50 and continuous positive airway pressure of 5 cm H_2_O, without inspiratory pressure augmentation, was unsuccessful
*t* = −60 h	**Start of structured respiratory monitoring window**	Mechanically ventilated and not yet ready for extubation	Pre-treatment respiratory monitoring period	Continuous EAdi monitoring, together with tidal volume and peak inspiratory pressure measurements, was analyzed from this time point onward
*t* = 0 h	**Initiation of pyridostigmine**	Persistent respiratory muscle weakness during mechanical ventilation	Median EAdi at treatment initiation was 1.69 [1.25–2.86] µV	Pyridostigmine was started. At this time point, median peak inspiratory pressure was 15.8 [15.3–24.2] cm H_2_O and tidal volume was 5.97 [5.37–6.44] mL/kg under pressure control 14 cm H_2_O, PEEP 6 cm H_2_O, and FiO_2_ 30%
*t* = 36 h	**Pyridostigmine dose escalation**	Early clinical improvement	—	Pyridostigmine dose was increased; continuous monitoring of EAdi, tidal volume, and peak inspiratory pressure was continued
*t* = 0 to 120 h	During pyridostigmine treatment and EAdi-guided respiratory monitoring	Progressive clinical improvement with recovery of respiratory muscle function	Median EAdi increased to 7.7 [5.12–11.07] µV during the observation period	Ventilator support could be reduced to pressure control 10 cm H_2_O, PEEP 5 cm H_2_O, and FiO_2_ 25%, with improvement in tidal volume and reduction in peak inspiratory pressure
Day 5 of treatment (*t* = 120 h)	End of treatment observation period	Clinically stable and considered ready for extubation	—	Successfully extubated without the need for additional respiratory support
Day 6	After completion of EAdi analysis	Stable after extubation	—	Transfer from the PICU to the pediatric neurology ward
2 weeks after admission	Post-PICU recovery	Continued clinical recovery	—	Discharged home on pyridostigmine without respiratory support
6-month follow-up	Long-term follow-up	Clinically stable, with only mild residual ptosis and no hospital readmissions	Repeat acetylcholine receptor antibody testing was positive, confirming the diagnosis of myasthenia gravis	Continued outpatient follow-up and pyridostigmine treatment; no recurrent respiratory insufficiency reported

**Figure 1:**
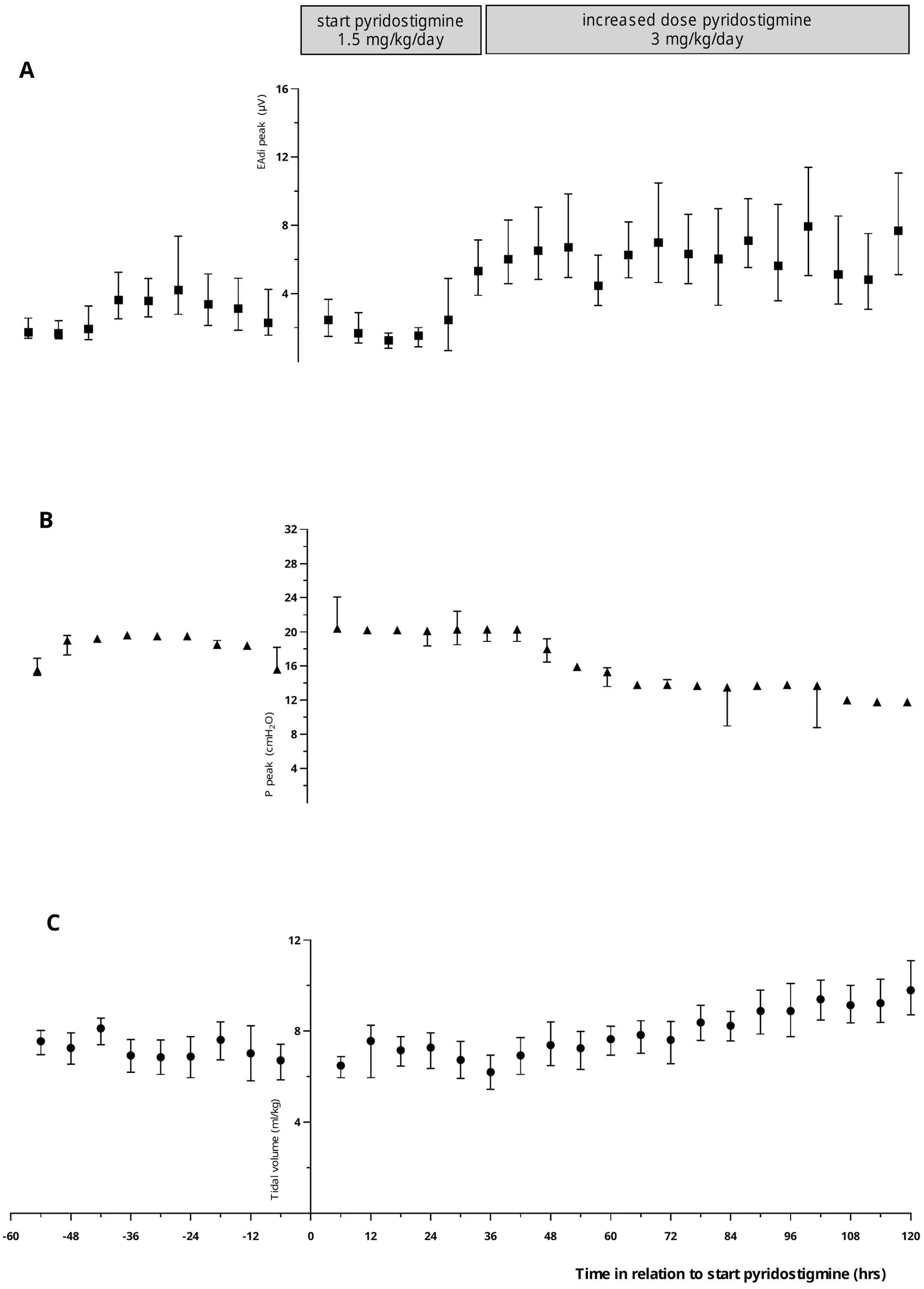
Time course of respiratory monitoring parameters in relation to pyridostigmine treatment. Median [IQR] values of **(A)** electrical activity of the diaphragm (EAdi), **(B)** peak inspiratory pressure (Ppeak), and **(C)** Tidal volume are shown over time. The structured respiratory monitoring window started at *t* = −60 h. *t* = 0 h indicates initiation of pyridostigmine, and *t* = 36 h indicates pyridostigmine dose escalation. The patient was successfully extubated at *t* = 120 h (day 5 of treatment). EAdi was monitored continuously using an esophageal electrode catheter, and values were summarized at 6 h intervals.

## Follow-up and outcomes

After 5 days of treatment the patient was successfully extubated without the need for additional respiratory support. The following day, after a total PICU stay of 10 days, she was transferred to the pediatric neurology ward. Two weeks later she was discharged home. At 12 months of follow-up she was still clinically stable with mild residual ptosis and she had not experienced any episode of respiratory insufficiency. Repeat testing at follow-up showed positive acetylcholine receptor antibodies, establishing a definitive diagnosis of early-onset myasthenia gravis and excluding congenital myasthenic syndrome as the underlying diagnosis.

## Patient perspective

A caregiver perspective was not available.

## Discussion

This case illustrates the potential value of monitoring EAdi as an adjunct to conventional ventilatory parameters in a child with neuromuscular respiratory weakness. EAdi provides bedside information on diaphragmatic activation that is not captured by airway pressure or tidal volume alone, and may therefore be useful when weakness is suspected during ventilator weaning.

In our patient, EAdi increased after initiation and escalation of pyridostigmine while ventilatory support could simultaneously be reduced, followed by successful extubation. This possible association suggests that EAdi captured recovery of neuromuscular transmission that was not yet visible in standard ventilator variables showed, pointing to a genuine treatment effect rather than a change driven by the ventilator settings themselves. This is particularly relevant here, as the patient's diagnosis was later revised from congenital myasthenic syndrome to early-onset myasthenia gravis, two conditions with different underlying pathophysiology and treatment response, which should be considered when generalizing our findings.

Koyama et al. reported a similar pattern in a patient with myasthenic weakness, where EAdi proved more informative than maximal inspiratory pressure in tracking clinical improvement (3). Diao et al. and Krishnakumar et al. have each described how diaphragm function assessment—including the Edi-peak/Vt ratio—can characterize respiratory muscle efficiency during spontaneous breathing trials and guide weaning decisions more broadly (1, 2). As suggested by the reviewers, we similarly calculated the Vt/EAdi ratio in our patient; its decline over the treatment period likely reflects a shift in the relative contribution of ventilator support vs. patient effort, in line with these prior observations. However, because pressure control and PEEP were intentionally adjusted during this period, we considered the ratio difficult to interpret in isolation in a single-patient case and chose not to present it as a separate figure. Krishnakumar et al. similarly emphasized the broader utility of diaphragm function assessment in guiding weaning in neuromuscular disease (1). Our case extends these observations to a young child with autoimmune myasthenic disease, in relation to pharmacological treatment rather than weaning alone.

This single-patient report has clear limitations. Normal pediatric EAdi reference values are not established and the Vt/EAdi ratio could not be robustly interpreted given concurrent ventilator changes. At the same time, EAdi should be interpreted cautiously and in clinical context, as it reflects neural drive rather than respiratory muscle strength alone and may be influenced by ventilator settings, sedation, comfort, and disease evolution. Therefore, this case does not support the use of EAdi as a standalone marker for extubation readiness, but rather as a complementary monitoring tool in children with suspected neuromuscular respiratory compromise.

## Data Availability

The original contributions presented in the study are included in the article/Supplementary Material, further inquiries can be directed to the corresponding author.
